# Electrophysiological Characteristics of Globus Pallidus Neurons

**DOI:** 10.1371/journal.pone.0012001

**Published:** 2010-08-06

**Authors:** Jenia Bugaysen, Maya Bronfeld, Hadass Tischler, Izhar Bar-Gad, Alon Korngreen

**Affiliations:** 1 The Leslie and Susan Gonda Multidisciplinary Brain Research Center, Bar-Ilan University, Ramat-Gan, Israel; 2 The Mina and Everard Goodman Faculty of Life Sciences, Bar-Ilan University, Ramat-Gan, Israel; Tokyo Medical and Dental University, Japan

## Abstract

Extracellular recordings in primates have identified two types of neurons in the external segment of the globus pallidus (GPe): high frequency pausers (HFP) and low frequency bursters (LFB). The aim of the current study was to test whether the properties of HFP and LFB neurons recorded extracellularly in the primate GPe are linked to cellular mechanisms underlying the generation of action potential (AP) firing. Thus, we recorded from primate and rat globus pallidus neurons. Extracellular recordings in primates revealed that in addition to differences in firing patterns the APs of neurons in these two groups have different widths (AP_ex_). To quantitatively investigate this difference and to explore the heterogeneity of pallidal neurons we carried out cell-attached and whole-cell recordings from acute slices of the rat globus pallidus (GP, the rodent homolog of the primate GPe), examining both spontaneous and evoked activity. Several parameters related to the extracellular activity were extracted in order to subdivide the population of recorded GP neurons into groups. Statistical analysis showed that the GP neurons in the rodents may be differentiated along six cellular parameters into three subgroups. Combining two of these groups allowed a better separation of the population along nine parameters. Four of these parameters (F_max_, AP_amp_, AP_hw_, and AHP_s_ amplitude) form a subset, suggesting that one group of neurons may generate APs at significantly higher frequencies than the other group. This may suggest that the differences between the HFP and LFB neurons in the primate are related to fundamental underlying differences in their cellular properties.

## Introduction

The external segment of the globus pallidus (GPe) is an intrinsic nucleus in the indirect pathway of the basal ganglia and is crucial to controlling their output [1], [2], [3]. The GPe receives GABAergic input from the striatum and glutamatergic input from the subthalamic nucleus (STN) [4], [5]. It sends GABAergic projections to the STN, internal segment of the globus pallidus (GPi) and the substantia nigra pars reticulata. Extracellular recordings in awake primates have revealed two types of GPe neurons: high frequency firing neurons with spontaneous pauses (HFP) and low frequency firing neurons with spike bursts (LFB) [3], [6]. Recordings from human patients undergoing surgery have shown similar groups [7]. It is still unknown whether cellular properties or different network connectivity account for these two types of neurons.

Surprisingly, the GPe of primates and humans are homologues of the globus pallidus (GP) in rodents [4]. In rodents, extracellular recording studies have also differentiated two types of GP neurons based on their specific waveforms and responses to apomorphine [8]. These GP neurons may also be classified into three groups by various membrane properties, such as membrane sag induced by hyperpolarization activated inward current (I_h_), rebound firing, spike accommodation, spike frequency adaptation and spike afterhyperpolarization [9], [10], [11]. However, anatomical studies in the rat provide conflicting data, one study reporting three types of GP projection neurons [12], while later studies found only two types of GP projection neurons [13], [14]. Recently, a combined physiological and computational study has suggested that the properties of GP neurons form a continuous space without differentiation into distinct groups or subgroups [15]. Thus, the existing evidence fails to provide a consensus as to the division of GP neurons into subgroups.

The current study aimed to test whether properties of cells recorded extracellularly in the primate GPe can be linked to cellular mechanisms underlying the generation of AP firing. We compared extracellular recordings in the behaving primate with *in vitro* recordings in the rat. This approach, drawing parallels between the cells in the rat GP and the primate GPe, enabled us to investigate possible neuronal sub-groups and the sources of their physiological characteristics.

## Results

**Figure 1:**
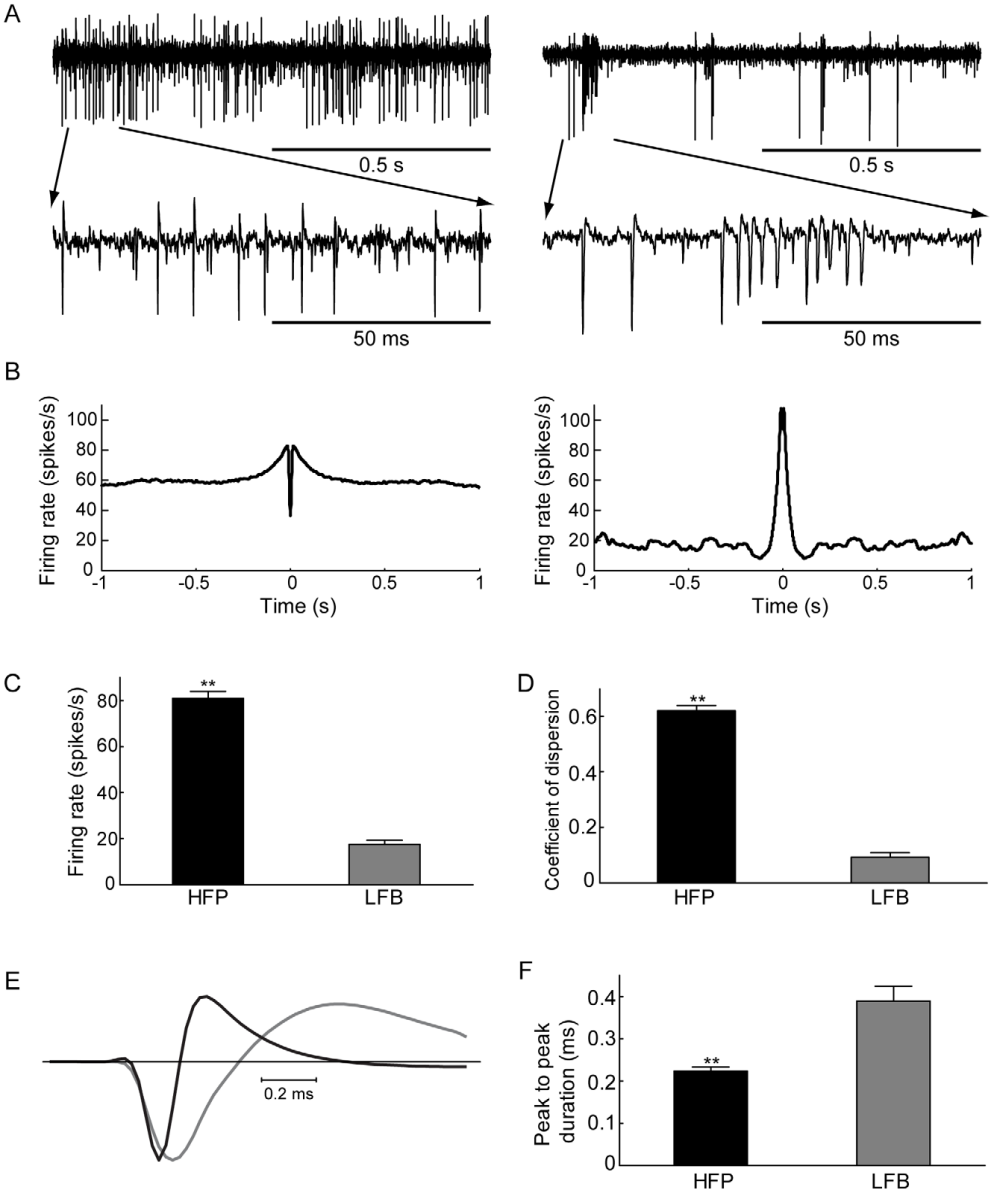
Extracellular properties of different cellular populations of the primate GPe. **A**. Traces from representative neurons from the two major neuron types in the GPe: high frequency pauser (HFP–left) and low frequency burster (LFB–right), shown at long and short time scales (top, 1 s trace; bottom, 100 ms trace). **B**. Autocorrelation functions of the neurons in A (maximal offset ±1 s). **C**. Mean firing rate of the two groups. **D**. Mean ISI distribution coefficient of dispersion of the two groups. **E**. Mean spike shape of the neurons shown above (HFP–black, LFB–gray). **F**. Mean spike duration of the two groups, Error bars indicate SEM, ** p≪0.01 Mann-Whitney U-test.

LFB and HFP neurons differ considerably in their firing patterns [3]. Can they be differentiated by other parameters? To answer this question we recorded extracellularly in the GPe of two primates. The spike shapes of two different neuronal populations, LFB (n = 12) and HFP (n = 47) neurons, were analyzed. LFB neurons are characterized by a low baseline firing rate with intermittent short bursts of high frequency spikes (Fig. 1A, right). HFP neurons are characterized by a high rate of irregular firing interspersed with pauses (Fig. 1A, left). Their firing properties are reflected in the very different autocorrelation function describing the neuronal activity of both groups (Fig. 1B). There was a significant difference (p≪0.01 Mann-Whitney U-test) in both firing rate (HFP: 66.5±6.0 spikes/s, LFB: 17.5±1.7, mean ± SEM, Fig. 1C) and the coefficient of dispersion, defined by variance (inter-spike-interval (ISI) distribution) /mean (ISI distribution) (HFP: 0.61±0.06, LFB: 0.09±0.02, mean ± SEM, Fig. 1D). The shape of the action potential varied between the recorded neurons (Fig. 1E). The mean width of the LFB neuron spikes (0.39±0.03 ms, mean ± SEM) was significantly larger than that of the spikes in the HFP group (0.21±0.01 ms, mean ± SEM, p≪0.01, Mann-Whitney U-test). Narrow APs are typical of neurons displaying high frequency firing. Thus, it is not surprising to observe this difference between the LFB and HFP neurons. This measure of AP width is shown below to be a good correlate of the intracellular AP width.

**Figure 2:**
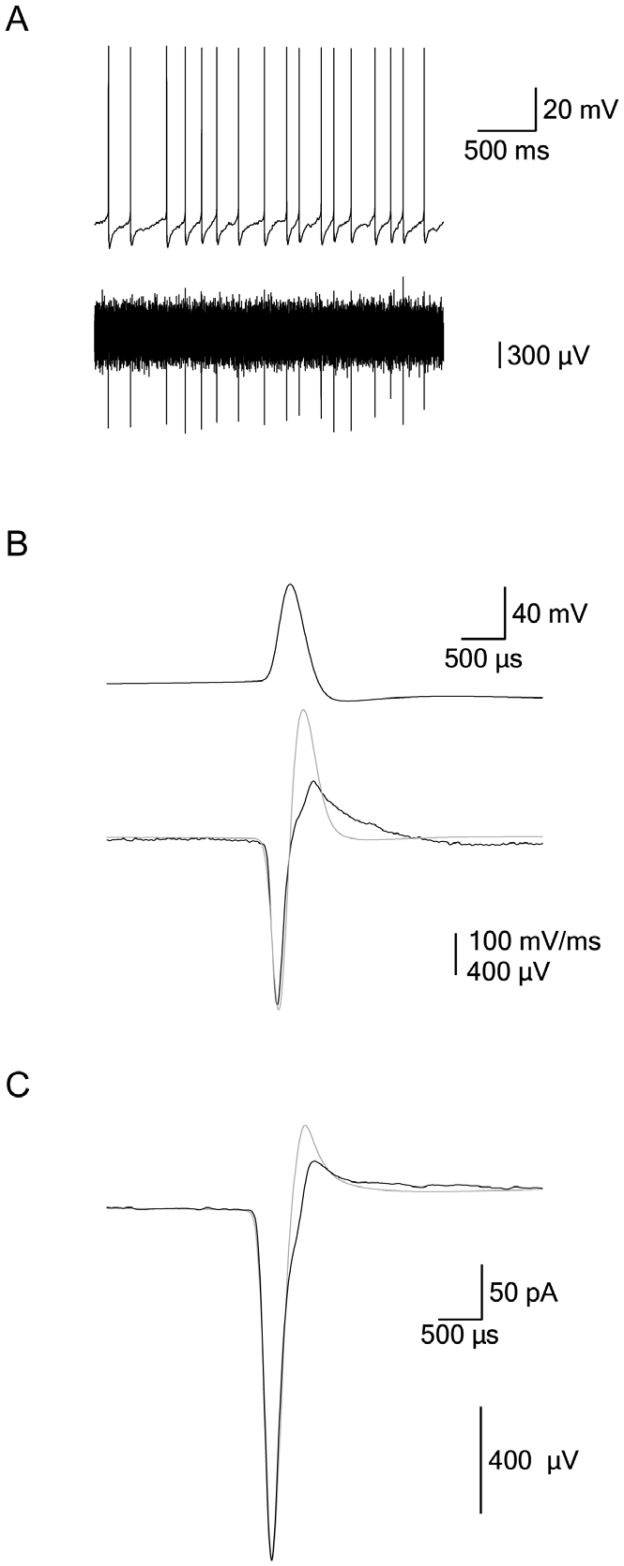
Intracellular and extracellular recordings from rat GP neurons. **A**, A continuous recording of the membrane potential recorded in the whole-cell mode of the patch-clamp technique (top trace) simultaneously with the extracellular potential (bottom trace). **B**, Spike-triggered average of the membrane potential during an AP (top trace) and the extracellular potential (bottom trace). The first derivative of the AP was superimposed on the extracellular potential (gray line). **C**, Event-triggered average of the voltage-clamp membrane current recording from a cell-attached patch (gray line) superimposed on the average of the extracellular potential (black line). All traces were filtered at 5 kHz and sampled at 20 kHz.

The recordings from primates suggested that the neurons in the GPe could be separated into two groups by the width of their AP. Furthermore, they suggested that these two populations differ in cellular properties that underlie the ability to produce high frequency firing. Since intracellular recordings from primates are not practical, it was decided to test this suggestion in an *in vitro* slice preparation from a rat, relying on the great similarity between the rat GP and primate GPe [4], [5]. It was clear to us that it may be problematic to relate extracellular properties recorded from primates to intracellular properties recorded in rats. To partially address this difficulty we first simultaneously recorded the membrane potential and the extracellular potential in acute rat brain slices (Fig. 2). We obtained clear extracellular recordings of the action potential when the extracellular electrode was positioned within ∼30 µm from the soma of the neuron currently being recorded intracellularly (Fig. 2A). At distances greater than ∼30 µm, the signal-to-noise ratio became too small to detect the extracellular potential.

To enable comparison between the intracellular and extracellular recordings we opted for spike-triggered averaging of the extracellular spikes using the intracellular APs as time markers. The shape of the average extracellular spike closely resembled the first derivative of the intracellular AP (Fig. 2B). There was also considerable similarity between the extracellular potential and the current flowing at the soma, recorded using cell-attached recordings in the voltage-clamp mode (Fig. 2C). Similar results were obtained from five further simultaneous recordings.

**Figure 3:**
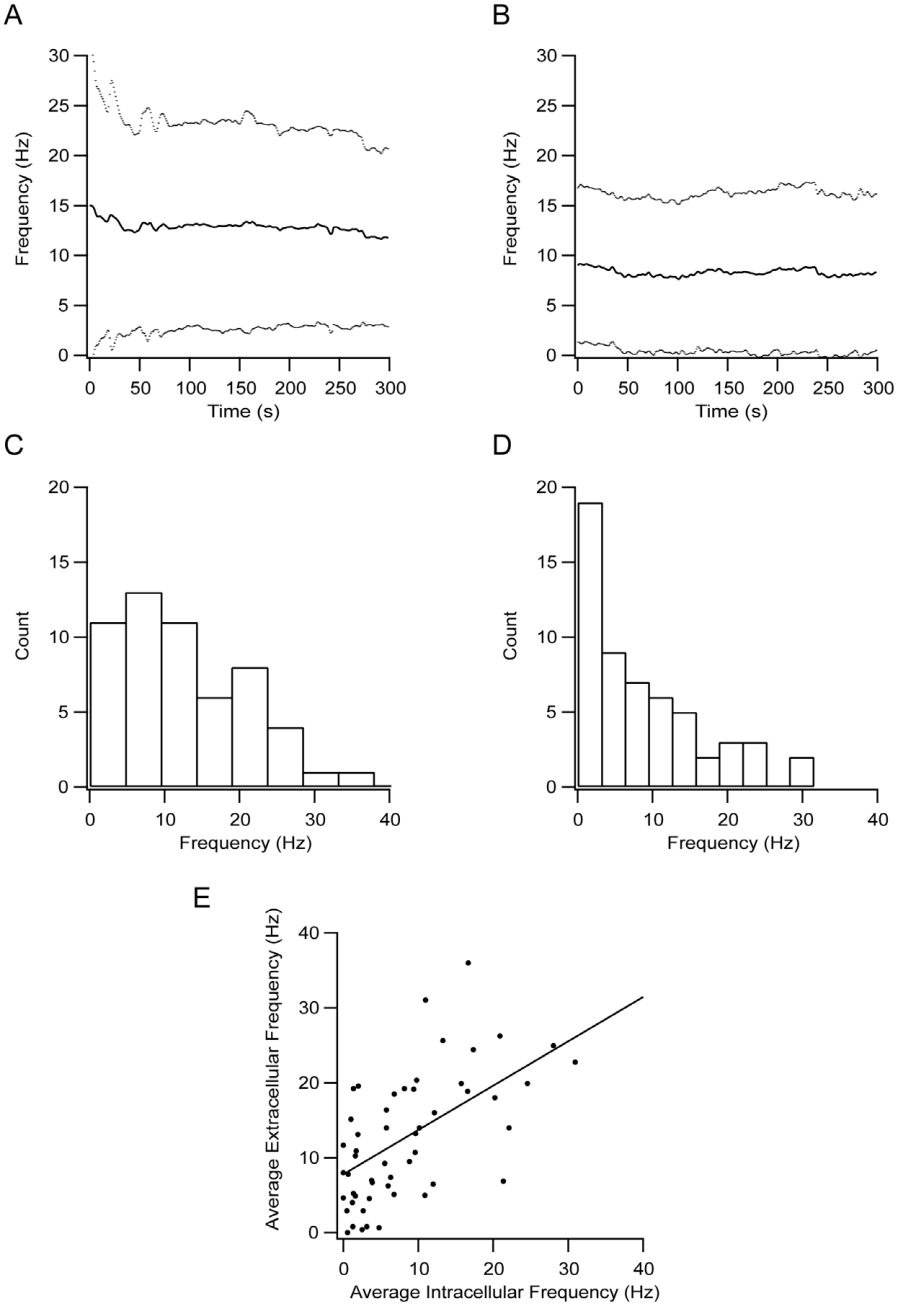
Spontaneous firing of GP neurons in acute rat brain slices. **A**, Population average of the spontaneous firing frequency recorded from GP neurons in the cell-attached mode (n = 76). **B**, Population average of the spontaneous firing frequency recorded from GP neurons in the whole-cell mode (n = 76). **C**, Distribution of the average firing frequency recorded in the cell-attached mode across the population. The frequency value used to construct the histogram was taken 200 s after the start of the recording to ensure stability. **D**, Distribution of the average firing frequency recorded in the whole-cell mode across the population. The frequency value used to construct the histogram was taken 200 s after the start of the recording to ensure stability. **E**, Correlation between the values used to generate C and D.

Given the similarity between the transmembrane current and the extracellular potential, we carried out all further experiments using the cell-attached configuration rather than an extracellular electrode. This substantially simplified the experiment. Recordings from each cell were made first in the cell-attached mode and then in the whole-cell mode. As previously reported, the spontaneous firing of GP neurons appeared to be regular [8], [16]. To quantify this, we recorded continuously for 5 minutes, extracted the spike timings, and calculated the instantaneous firing frequency and coefficient of variation (CV) from the ISI. The instantaneous firing rate displayed stable values and the CV was significantly smaller than 1 (Table 1), indicating a highly regular spike generation process. This analysis was repeated for all 76 neurons recorded. The mean firing frequency of the entire population, measured in each cell immediately after obtaining the cell-attached recording, was 15±15 Hz (n = 76). This decayed to a stable level of 13±10 Hz within 2 minutes of recording (Fig. 3A). The average spontaneous frequency recorded in the whole-cell mode was stable at 9±8 Hz during the entire 5 minutes of recording (Fig. 3B). Similar firing frequencies have been observed in in-vivo recordings from rats [8]. Frequency histograms from both extracellular (Fig. 3C) and intracellular recordings show the variability of the spontaneous firing frequency in the recorded neurons (Fig. 3D). Both histograms displayed similar trends, with most of the recordings clustered at frequencies below 10 Hz. Despite the similarity between the histograms, the intracellular and extracellular firing frequencies recorded from the same cells were only weakly correlated (Fig. 3E).

**Table 1:** Electrophysiological properties of GP cells.

*Properties*		*Type A (n = 14)*	*Type B (n = 24)*	*Type C (n = 38)*
**Intracellular parameters**				
Spontaneous firing rate	Hz	4±3	17±7	11±7
STD of spontaneous firing rate	Hz	1±0.3	2.3±1	1.6±0.8
CV of spontaneous firing rate		0.6±0.4	0.2±0.1	0.3±0.2
Fano factor of spontaneous firing rate	ms	0.4±0.3	0.4±0.2	0.3±0.3
V_m_	mV	−54±4	−53±4	−54±4
R_in_	MΩ	306±72.5	260±36.5	217±48.5
Sag	mV	42±8	18±7	23±12
Sag ratio		0.7±0.1	0.8±0.1	0.8±0.1
F_max_	Hz	108.5±53	266.6±170	121±48.5
Current inducing 63% of F_max_	pA	288.5±246	521.5±513	319±256
Monophasic AHP		+	―	+
Biphasic AHP		―	+	―
AHP_s_ amplitude	mV	18±2	14±3	16±3
AHP_f_ amplitude	mV	N/A	13±4	N/A
AP adaptation ratio		0.5±0.1	0.65±0.1	0.75±0.1
AP_amp_	mV	80±7	69±8	77.5±9
AP_hw_	ms	0.6±0.1	0.4±0.15	0.5±0.1
AP threshold	mV	−42±5	−44±5	−43±4.5
Rebound firing		―	+	―
Firing pattern		irregular	regular	regular
**Extracellular parameters**				
Spontaneous firing rate	Hz	8±5	14±6	12±10
STD of spontaneous firing rate	Hz	1.3±0.5	2.2±1	1.6±0.7
CV of spontaneous firing rate		0.3±0.2	0.2±0.1	0.3±0.2
Fano factor of spontaneous firing rate	ms	0.3±0.2	0.4±0.3	0.3±0.2
AP_ex_ duration	ms	0.8±0.2	0.5±0.1	0.7±0.2

Data are expressed as mean ± S.D. *n* =  number of recorded GP neurons in each group.

Although spontaneous firing frequency measured intracellularly and extracellularly appeared similar, their weak correlation allows only partial correlation of intracellular and extracellular firing. Since the recordings from primates suggested that the population of neurons could be divided using the width of the AP, we further characterized the relation between the width of the intracellular and extracellular potentials in the rat.

**Figure 4:**
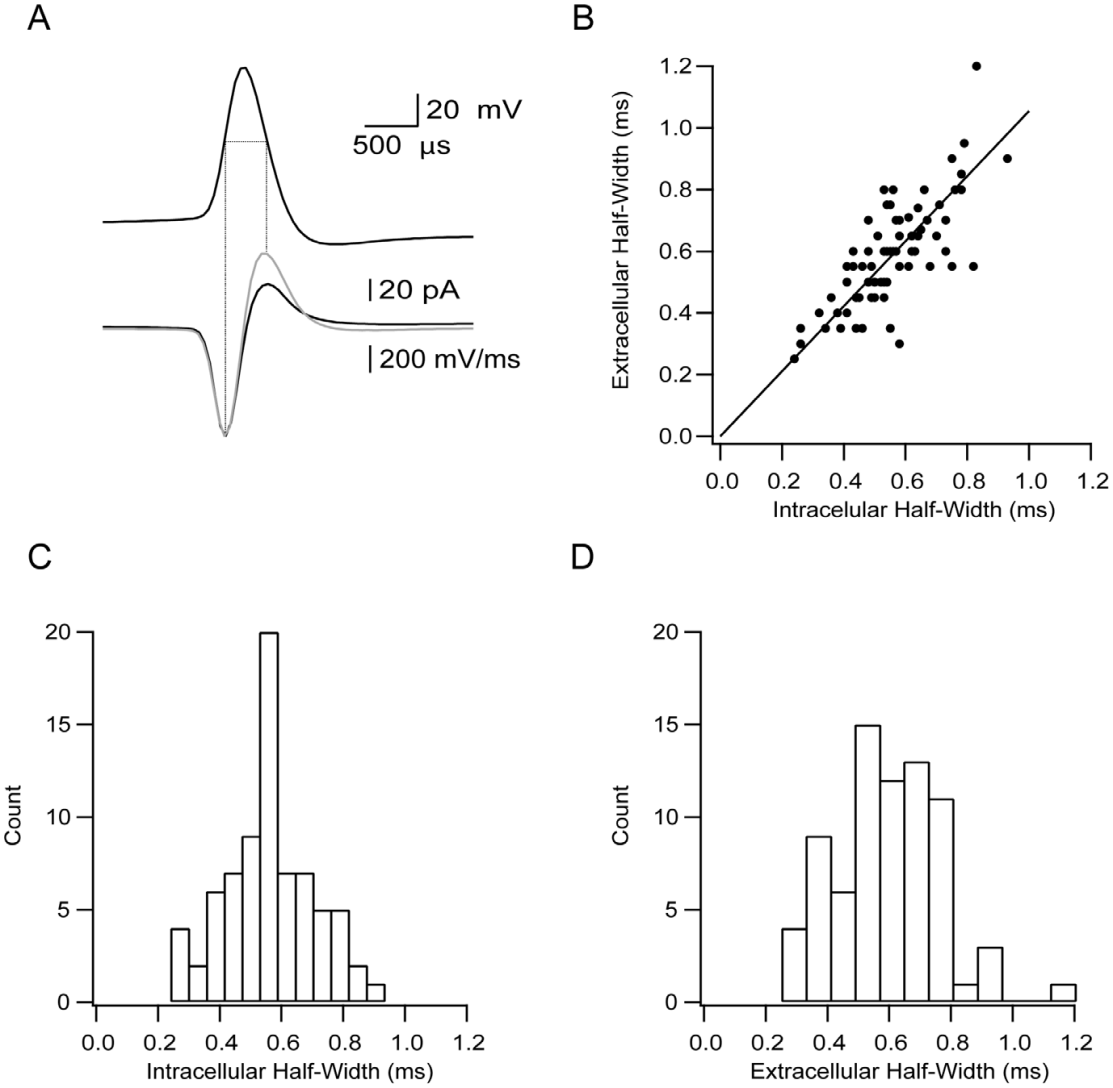
The width of the intracellular AP can be extracted from extracellular recordings. **A**, The intracellular AP recorded in the whole-cell mode (top trace) and the transmembrane current recorded in the cell-attached mode (bottom trace, black line). The first derivative of the intracellular trace is superimposed on the extracellular trace (bottom trace, gray line). The half-width of the intracellular AP is indicated by the horizontal line. The vertical lines locate the half-width of the intracellular AP on the extracellular trace. **B**, Correlation between the half-width of the extracellular and intracellular recordings of the AP for all neurons recorded (n = 76). **C**, Distribution of the intracellular AP half-width for all neurons recorded. **D**, Distribution of the extracellular AP half-width for all neurons recorded.

The half-width of the intracellular AP (AP_hw_) was calculated by measuring the width of the AP at the midpoint between threshold and peak. The half-width of the intracellular AP could also be extracted from the first order numerical derivative of the intracellular AP by measuring the time delay between the minimum and maximum values of this derivative (Fig. 4A). As previously observed [17] and predicted by numerical simulations [18], the shape of the somatic membrane current closely resembled that of the first derivative of the intracellular AP. The widths of the extracellular and intracellular APs were highly correlated (Fig. 4B, R ^2^ = 0.82).

Can the AP half-width recorded in rats be used as a parameter to dissect the neuronal populations of the GP into groups as in primate? To investigate this we constructed histograms for the intracellular (Fig. 4C) and extracellular (Fig. 4D) AP half-widths. Both histograms were unimodal and visual inspection showed no clear separation of the recorded pool of neurons into subgroups. Can other intracellular or extracellular parameters measured in acute brain slices be used to sort the population into groups? It has been suggested that GP neurons can be classified into distinct functional groups by visual inspection and by variable membrane properties [9], [10], [11]. Therefore, the lack of clear separation into groups using the AP half-width may have resulted from experimental noise or the developmental stage of the rats. To overcome these potential problems we extracted additional parameters characterizing the firing modes of GP neurons. The responses of GP neurons to varying levels of current injection via a whole-cell pipette were used to extract the size of the sag of the membrane potential, the rebound firing, the mono- or biphasic AHP, the pattern of firing in response to injected depolarizing currents, and the AP adaptation ratio. We then attempted to dissect the population using these parameters and applying visual analysis similar to those used previously [9], [10], [11]

**Figure 5:**
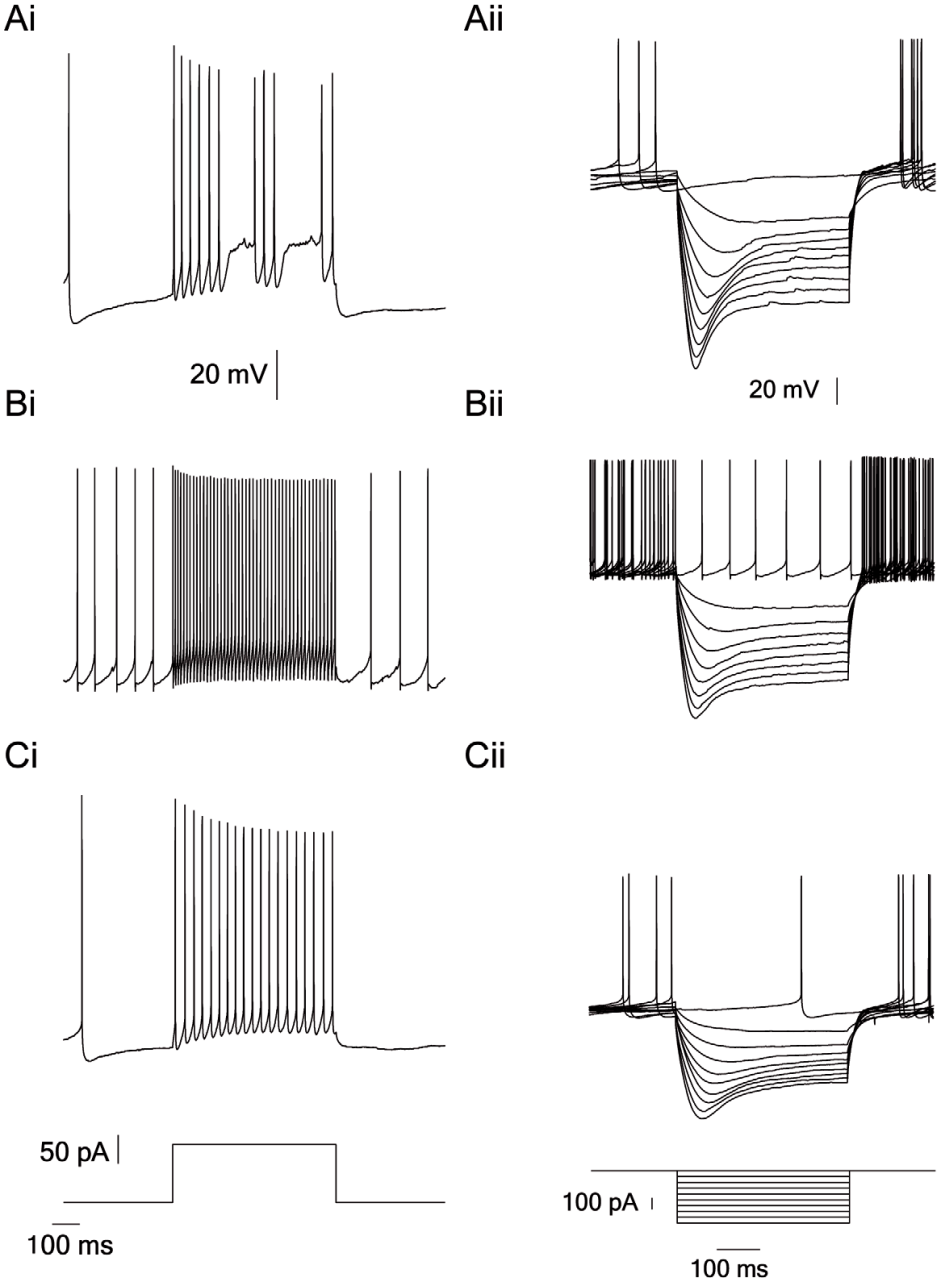
Representative recordings from GP neurons of three visually separated subgroups. *Ai*, *Bi*, and *Ci*, responses of GP cells to depolarizing current steps (50 pA increment, 600 ms) applied from 0 to 550 pA using whole-cell configuration of the patch-clamp technique. Representative membrane potentials recorded in response to 100 pA are given for each cell type. Sampled at 20 kHz and filtered at 10 kHz. *Aii*, *Bii*, and *Cii*, responses of GP cells to hyperpolarizing current steps applied from 0 to −450 pA (50 pA increment, 600 ms) using the whole-cell configuration of the patch-clamp technique.

The visual separation allowed us to divide the population into three groups. One group of neurons, referred to as type A neurons, responded to depolarizing current injection with burst firing (Fig. 5Ai). Type B neurons responded to the same depolarizing stimulus with high frequency regular firing (Fig. 5Bi), while type C neurons responded with a slower firing frequency (Fig. 5Ci). Type B neurons displayed biphasic after-hyperpolarization (AHP, Fig. 5Bi and 5Bii), while the other two types displayed only a slower monophasic AHP. In addition, visual inspection suggested that the sag of type A neurons was larger than in the other two groups (cf. Fig. 5Aii and Fig. 5Bii, 5Cii). Finally, the type B neurons displayed smaller AP adaptation following depolarization (Fig. 5Bi).

**Figure 6:**
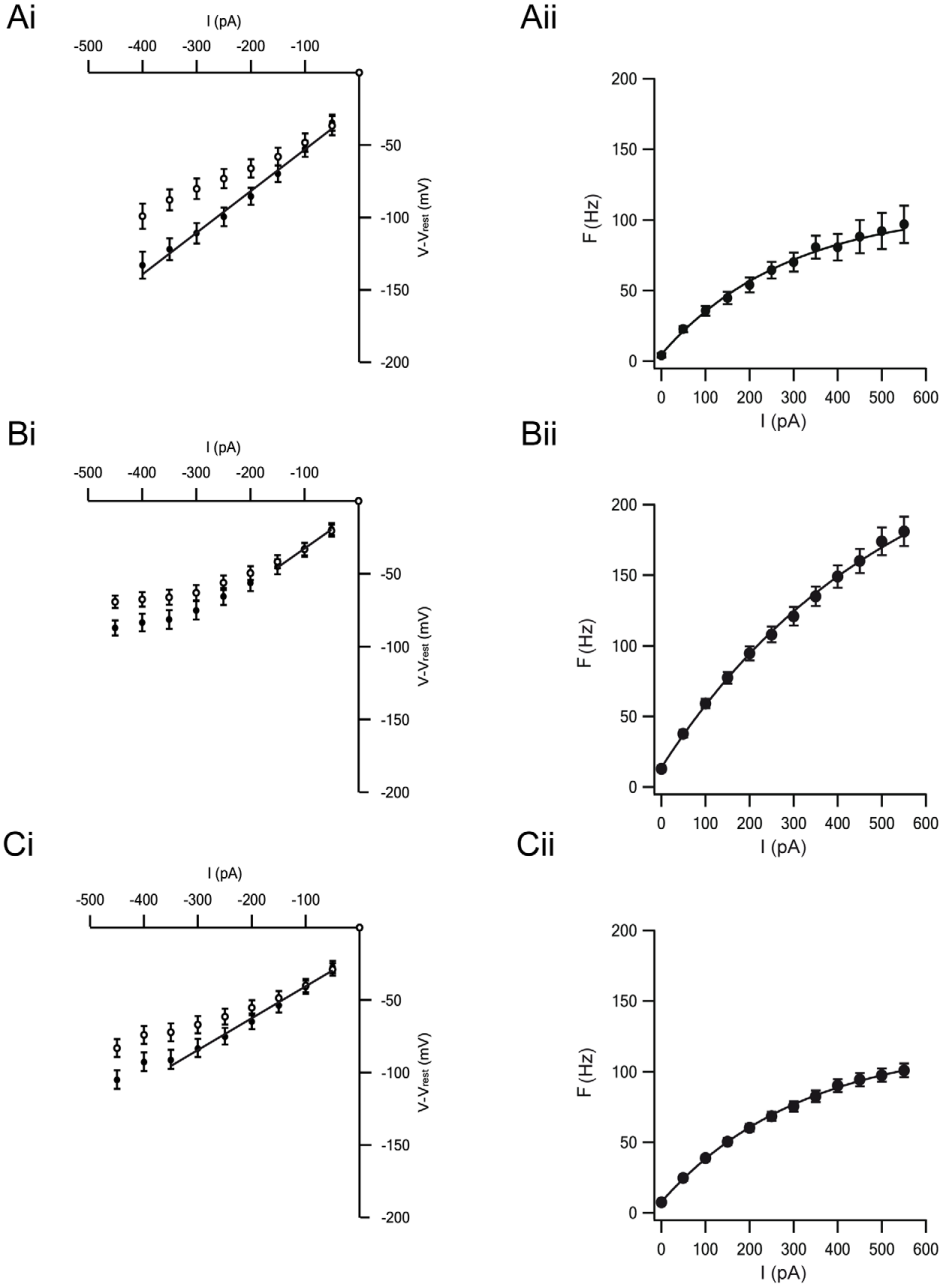
Average parameters of GP neurons. *Ai*, Voltage-current curves for type A neurons (n = 14). *Bi*, Voltage-current curves for type B neurons (n = 24). *Ci*, Voltage-current curves for type C neurons (n = 38). In Ai, Bi and Ci, • - data measured to calculate input resistance; ○ - data measured to estimate the influence of I_h_ on membrane potential. *Aii*, lines plotted by exponential fitting to curves from action potential frequency vs. injected currents. F_max_ and current required to reach 63% of maximal firing rate were extracted by exponential fit for type A neurons. Values and error bars are mean ± S.E. Bii, As in Aii but for type B neurons. Cii, As in Aii but for type C neurons.

To further investigate the differences between the three proposed groups we quantified the sag of the membrane potential and calculated the R_in_ of each cell type from current-voltage (I–V) curves of the neurons (Fig. 6). The I–V curves were extracted from the maximal deflection of the membrane potential following hyperpolarizing current injection and from the stable membrane potential level reached at the end of the pulse. It was predicted that the maximal deflection in the membrane potential would be linearly dependent on the injected current since I_h_ was still not activated. In fact, for types A and C the I–V curve was linear over the entire the current range applied in this study (Fig. 6Ai and 6Ci). However, the I–V curve for type B neurons was non-linear, suggesting the involvement of an additional rectification mechanism (Fig. 6Bi). All the I–V curves calculated from the steady-state value of the membrane potential displayed substantial rectification as would be expected from the activation of I_h_. The parameters extracted from these curves are reported in Table 1.

**Figure 7:**
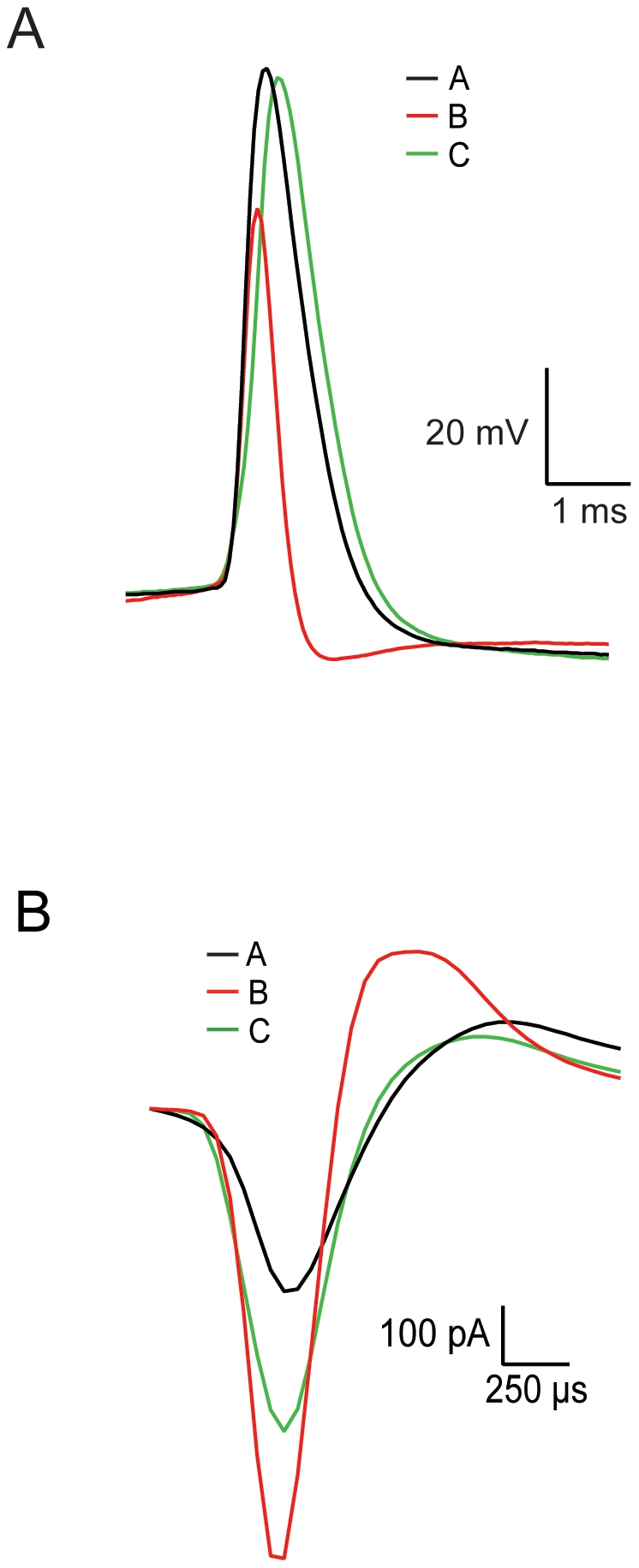
Representative intracellular and extracellular APs. **A**, Spontaneous AP recorded in the whole-cell mode superimposed at an extended time scale for each cell type ( ─ type A; ─ type B; ─ type C). **B**, Spontaneous AP recorded in cell-attached mode superimposed at an extended time scale for each cell type (─ type A; ─ type B; ─ type C).

Next, we generated current-frequency (F–I) curves to analyze the firing activity of each cell type in response to depolarizing current injections (Fig. 6). Similar to the I–V curves, the F–I curve generated from group B neurons could be visually differentiated from those of the two other groups. The current required to induce firing at 63% of the maximal firing rate of type A, B, and C neurons was 288.5±246 pA (Fig. 6 Aii, n = 14), 521.5±513 pA (Fig. 6Bii, n = 24), and 319±256 pA (Fig. 6 Cii, n = 38), respectively. The maximal firing rate (F_max_) of type A, B, and C neurons was 108.5±53 Hz (n = 14), 266.6±170 Hz (n = 24), and 121±48.5 Hz (n = 38), respectively. Statistical analysis of these differences appears in Table 2. Another feature that could visually differentiate the three types was the shape of the AP (Figure 7). The AP of neurons from group B was smaller and narrower than that observed in the two other groups (Fig. 7A). This difference was also clear in the cell-attached recordings (Fig. 7B) and is reminiscent of the difference observed between the AP shape of primate LFB and HFP cells (Fig. 1B). These parameters and other extracellular and intracellular properties calculated in the present study are summarized in Table 1.

**Table 2:** Statistical analysis of the intracellular and extracellular physiological parameters of GP cells.

	*KW test*		*MWW test*		
	P	P (AvsB)	P (AvsC)	P (BvsC)	P (ACvsB)
**Intracellular Parameters**					
Spontaneous firing rate	3e-4*	8e-5*	0.006*	0.04*	0.002*
CV of firing rate	0.01*	0.002*	0.01*	0.7	0.1
Fano factor of firing rate	0.5	0.8	0.3	0.3	0.5
V_m_	0.2	0.1	0.9	0.08	0.06
R_in_	1e-5*	2e-6*	2e-5*	0.5	0.03*
Sag ratio	9e-4*	5e-5*	0.006*	0.2	0.02*
Sag	1e-8*	2e-9*	4e-7*	0.05*	5e-4*
F_max_	8e-8*	5e-6*	0.01*	4e-6*	2e-7*
Current inducing 63% of F_max_	0.01*	0.009*	0.004*	0.8	0.3
AP_amp_	1e-4*	2e-4*	0.4	3e-4*	4e-5*
AP_hw_	6e-6*	5e-5*	0.06	6e-5*	4e-6*
AHP_s_ amplitude	9e-4*	2e-4*	0.02*	0.05*	0.004*
AP threshold	0.7	0.4	0.6	0.9	0.7
AP adaptation ratio	2e-6*	0.01*	7e-7*	0.006*	0.2
**Extracellular parameters**					
Spontaneous firing rate	0.03*	0.009*	0.5	0.06	0.2
CV of spontaneous firing rate	0.9	0.8	0.8	0.99	1
AP_ex_ duration	3e-6*	0.02*	0.03*	3e-5*	1e-4*
Fano factor of spontaneous firing rate	0.2	0.1	0.3	0.2	0.1

Kruskal-Wallis one- way analysis of variance by ranks and Mann-Whitney-Wilcoxon tests were used for the intracellular and extracellular properties of GP cells.

*P-value≤0.05.

Visual dissection of the neuronal population may lead to selection bias. In addition, it was not clear whether the parameters used in the separation process (Table 1) allowed classification into three statistically distinct groups. We therefore used a Kruskal-Wallis one-way analysis of variance by ranks to statistically test the population of GP neurons based on these extracted properties (Table 2). The null hypothesis that the GP neuron population was not uniformly distributed was accepted for 14 of the measured parameters (p<0.05). We next used Mann-Whitney-Wilcoxon (MWW) tests to examine which parameters differentiated the GP neuron population into different subpopulations (Table 2). 13 of the measured intracellular parameters were significant in at least two pairs of subgroups. However, only six parameters (intracellular spontaneous firing rate, Sag, F_max_, AHP_s_ amplitude, AP adaptation ratio, and AP_ex_ duration) were significant for all three pairs of groups tested.

**Figure 8:**
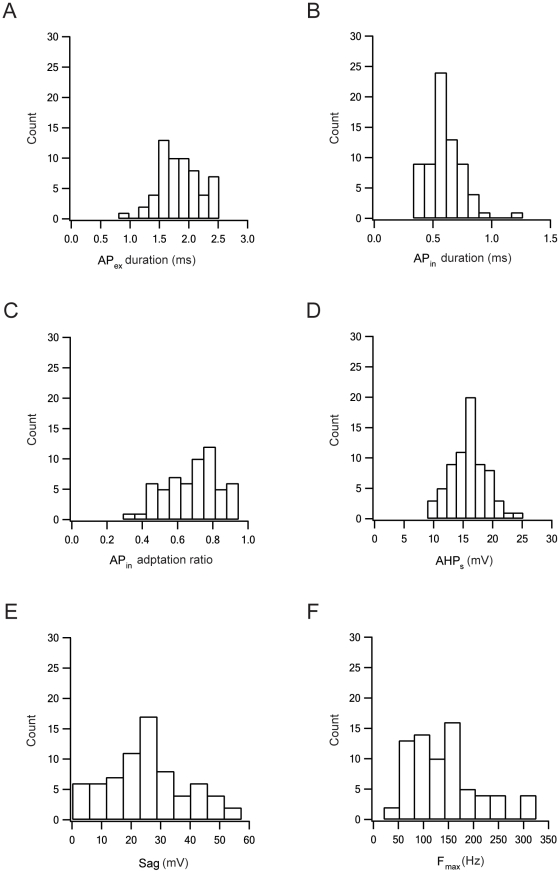
Histograms of several extracellular and intracellular properties of GP cells. ***A***, Half-width of the extracellular AP (AP_ex_) calculated as in figure 4. ***B***, Half-width of the intracellular AP (AP_in_) calculated as in figure 4. ***C***, The intracellular AP adaptation ratio calculated as the ratio between the amplitude of the last AP and that of the first AP in a train of APs generated by current injection. ***D***, The slow phase of the AHP (AHP_s_). AHP amplitude was measured from threshold. ***E***, Sag of membrane potential induced by activation of I_h_. Sag was calculated as the difference between the maximal deflection of the membrane potential following a hyperpolarizing step and the deflection at the end of the pulse. **F**, Maximal frequency calculated for each cell from F-I curves, similar to those in figure 6.

As noted above, there were clear differences between the I–V and F–I curves of group B and those of the two other groups (Fig. 6). In addition, group B displayed biphasic AHPs while the two other groups displayed only monophasic AHPs (Fig. 5 and Table 1). This latter observation suggested a possible separation criterion, since it presented a binary separation rule rather than a continuous one. We therefore combined the parameters of groups A and C into one group and re-applied the MWW test between this super- group and group B. Under these conditions the two groups could be significantly differentiated by 9 parameters (Table 2). Finally, to allow visual inspection of the distribution of the parameters we generated histograms from the extracellular and intracellular parameters obtained here. The six different histograms in figure 8 were chosen, based on their statistical significance, to create at least two subgroups in MWW tests (Table 2). Despite the inference drawn from the statistical analysis, that the population could be divided into several groups, none of the parameter distributions displayed a clear bi- (or multi-) modal structure. But they were not clearly unimodal either. Similar complex histograms were generated for the remaining parameters extracted in this study (data not shown). Thus, while the statistical analysis clearly indicated that the population was heterogeneous, it was not possible to conclude with any confidence that the GP neurons could be classified into two or three distinct neuronal types.

## Discussion

In this study we recorded from rat brain slices in order to determine whether the differences between LFB and HFP neurons in the globus pallidus of primates are due to cellular or to network properties. To do this, we compared intracellular and extracellular characteristics of the AP recorded from neurons in the rat globus pallidus and classified the population of recorded neurons into functional groups. We then used the properties of these groups to predict the cellular properties of neurons in the primate GPe.

Initially, we found that the extracellular width of the AP (AP_ex_) in behaving primate could differentiate the neurons in the two groups in the GPe. We then showed that the AP_ex_ in the rat could serve as a quantitative measure for the intracellular AP_hw_. Analysis of the response of GP neurons to current injection in the whole-cell mode suggested that the neurons in the rat could be divided into three groups. Statistical analysis of these groups revealed that six of the parameters (intracellular spontaneous firing rate, Sag, F_max_, AHP_s_ amplitude, AP adaptation ratio, and AP_ex_) significantly divided the population into three groups. Combining two groups into one super-group increased the number of parameters dividing the population to 9. These data from the rat thus hint that the neuronal population in the primate GPe may be composed of two neuronal types with different cellular properties.

The division of neurons in the primate GPe into LFB and HFP groups was established in the early days of basal ganglia electrophysiology [3]. This study characterized 85% of the neurons of the GPe as HFPs and the remaining 15% as LFBs. These numbers may be biased by the general tendency of experimenters to record high-frequency neurons rather than mostly quiescent neurons. Later studies demonstrated that LFBs not only differ from HFPs in terms of their firing rate and pattern, but also in their modulation by the state of the animal. Treatment of a primate with 1-methyl-4-phenyl-l,2,3,6-tetrahydropyridine (MPTP), rendering it parkinsonian, leads to a considerable decrease in firing rate and to an increase in the irregularity of HFPs, with a concomitant increase in firing rate of LFBs and an increase in regularity [19]. Injection of dopamine agonists leads to the reversed differential activation, exciting HFPs while completely inhibiting LFBs [20]. These results suggest that the different types of neurons may be differentially connected within the network and may play different roles in the function of the GPe. Equivalent results have been obtained *in vivo* in the rat, as two populations of neurons with different extracellular waveforms were found. The neurons of the two groups responded differentially to a dopamine agonist with type I reducing their firing rate and type II increasing their rate [8] similar to the responses of primate LFB and HFP neurons. Moreover, it has been previously reported that the morphological features of rodent pallidal neurons are correlated with their electrophysiological properties [11]. Thus, it may be possible to speculate that similar morphological differences may exist between HFP and LFB neurons in primates. These similarities suggest that dissecting out groups from the population of rat GP neurons can allow us to predict the cellular properties of the primate neurons.

Given the differences between the extracellular waveforms of primate LFB and HFP neurons (Fig. 1), it was clear that we needed to establish a correlation between the waveform of the extracellular and the intracellular potentials. This correlation was established in several stages, starting from simultaneous recordings of both signals (Fig. 2). Using a similar procedure to that of Henze et al. [17], we extracted the width of the AP from extracellular recordings (Fig. 4). The firing rate of the neurons recorded in our *in vitro* preparation was in the same range as that recorded in the behaving rat, which was 8–10 spikes/s, depending on the state of the animal [8], [16]. The average spontaneous firing frequency recorded in the cell-attached mode was only weakly correlated with that recorded in the whole-cell mode (Fig. 3E). Several factors may contribute to this weak correlation. The slow change in firing frequency in the cell-attached mode (Fig. 3A) may be related to the slow washout of residual potassium ejected from the patch pipette during gigaseal formation from the bathing solution. Second, the lower average firing frequency recorded in the whole-cell mode may be due to partial washout or rundown of ionic channels by the whole-cell solution. This possible channel rundown was probably small, since the width of the AP remained stable (Fig. 4). Small changes to conductances active around the resting membrane potential and participating in determining the spontaneous firing rate may cause large shifts in spontaneous firing rates without affecting the shape of the AP or the maximal firing frequency of the neuron.

Several methods have been used to classify populations of neurons into subgroups. In the GP these methods have mostly included visual inspection of the recorded data followed by extraction of visually salient parameters [9]. The consensus has been that the neuronal population in the GP can be divided into three types [9]. However, a large database was recently constructed using simulated surrogate data and the parameter distributions were compared to those observed experimentally [15]. This study suggested that the variability of the neuronal population in the GP was due to a continuous variation in the cellular properties of the neurons. Here we relied on the differences in the extracellular AP of LFB and HFP neurons in the primate to extract a relevant group of parameters for rat GP neurons that was then dissected with non-parametric statistical analysis. None of the parameter distributions displayed a clear bi- (or multi-) modal structure, nor were they clearly unimodal (Fig. 8). This could be due to experimental noise, large variability and overlap between the values of the parameters of the different groups. Alternatively, there could be only one highly variable group of neurons in the GP.

The parameters in our data did not display a normal distribution, thus we used non-parametric methods to assess the statistical differences among the groups. The division into the different groups was based on a few parameters and was verified by the division for other parameters. However, the separation of the data into groups using the parameters did not enable automatic clustering into either two or three groups. The data reside in a high dimensionality space derived from the number of parameters. The sparseness of the data points leads to the problem known as the “curse of dimensionality” [21], which prevented efficient clustering of the data into groups. Attempts to reduce the dimensionality by generating parameter sub-groups and principal components of the parameter space did not provide a useful substrate for clustering.

Thus, our current study does not provide a clear dissection of the GP population into groups, even though statistical analysis suggests that the population may be divided into 2–3 groups, unlike the database modeling suggesting that the population is homogenous [15]. It is clear that further research is needed. Nevertheless, our and other studies clearly show that some GP neurons respond to stimulation with higher firing frequencies than others. We show that the ability to generate higher firing frequency is related to the width of the AP and other cellular parameters (F_max_, AP_amp_, AP_hw_, and AHP_s_ amplitude). Furthermore, the width of the extracellular potential was significantly different between the HFPs and LFBs in the primate GPe. This hints that the observed firing modes of these two types of neurons in the primate GPe may be due to different cellular properties. Thus, there could be a basic mechanistic difference between them, possibly a differential expression of either voltage-gated sodium or potassium conductances. This possible mechanistic model may be important for research on the dynamic properties of the GPe. Furthermore, the parallels observed here between the properties of neurons in the rat GP and the primate GPe may help combine system level studies of the primate GPe with cellular studies in the rodent GP. It is important to note that, since recording intracellularly from neurons in primates is not possible, the differences in AP shapes may be partly induced by network activity.

## Methods

### *In vitro* slice preparation and solutions

Thick sagittal slices of 300 µm were obtained from rat somatosensory cortex, striatum and GP using previously described techniques [22]. Wistar rats, 12–21 days old, were killed by rapid decapitation according to the guidelines of the Bar-Ilan University animal welfare committee. This procedure was approved by the national committee for experiments in laboratory animals at the ministry of health (permit number BIU281206). The brain was quickly removed and placed in ice-cold artificial cerebrospinal fluid (ACSF) containing (in mM): 125 NaCl, 3.5 KCl, 25 NaHCO_3_, 1.25 Na_2_HPO_4_, 1.5 CaCl_2_, 1 MgCl_2_, 25 glucose and 0.5 Na-ascorbate (pH 7.4 with 95% O_2_/5% CO_2_). Slices were cut using an HR2 Slicer (Sigman Electronic, Germany) and transferred to a submersion-type chamber, where they were maintained for the remainder of the day. Experiments reported here were carried out at 34°C. The GP nucleus and individual GP neurons were visualized using infrared differential interference contrast (IR-DIC) microscopy. The recording chamber was constantly perfused with oxygenated ACSF. The standard pipette solution contained (in mM): 130 K-gluconate, 10 KCl, 10 HEPES, 4 MgATP, 10 Na-phosphocreatin, 0.5 EGTA and 0.3 GTP (Sigma) (pH 7.2 with KOH). The reference electrode was an Ag-AgCl pellet placed in the bath. The 10 mV liquid junction potential measured under the ionic conditions reported here was not corrected for.

### *In vitro* electrophysiology

Cell-attached and whole-cell recordings were obtained from the soma of GP neurons with an Axopatch-200B amplifier (Axon Instruments). Voltage was filtered at 10 kHz and sampled at 20 kHz, unless stated otherwise, using patch pipettes (4–8 MΩ) pulled from thick-walled borosilicate glass capillaries (2.0 mm outer diameter, 0.5 mm wall thickness, Hilgenberg, Malsfeld, Germany).

### *In vivo* experiments

*In vivo* data were obtained from two male cynomolgus monkeys (*Macaca fascicularis*). The monkeys' water, food consumption and weight were followed daily and their health was monitored by a veterinarian. All procedures followed the National Institutes of Health Guide for the Care and Use of Laboratory Animals, Bar-Ilan University Guidelines for the Use and Care of Laboratory Animals in Research and in accordance with the recommendations of the Weatherall Report. All procedure were approved and supervised by the Institutional Animal Care and Use Committee (IACUC). This procedure was approved by the national committee for experiments in laboratory animals at the ministry of health (permit number BIU220506). Full details of the surgery and recording procedures appeared elsewhere [23]. Briefly, the monkeys underwent a surgical procedure to attach a recording chamber to the skull allowing access to the GPe. Recording sessions began after recovery from surgery. The monkeys were seated in a primate chair with their head fixed during the recording sessions. Using a cylindrical guide, eight glass-coated tungsten microelectrodes (impedance 0.2–0.7 MΩ at 1 kHz) were advanced separately into the GP. The electrode signal was continuously sampled at 40 kHz (Alphamap 10.10, Alpha–Omega Engineering), amplified (X1000) and wide bandpass filtered (2–8000 Hz four-pole Butterworth filter) (MCP-Plus 4.10, Alpha–Omega Engineering). The external (GPe) and internal (GPi) segments were distinguished online based on characteristics of neuronal activity and the existence of border cells and white matter between the two segments. Only high-frequency pausers (HFP) and low-frequency bursters (LFB) from the GPe were included in this study.

### Data analysis

All off-line analysis was carried out with Offline Sorter 2.8.6 (Plexon), NeuroExploler 4.007 (Nex Technologies), Matlab R2007b (Mathworks) and IgorPro 5.0 (WaveMetrics) on a personal computer. The frequency of spontaneous extracellular and intracellular firing was calculated from spikes extracted from 5 min of continuous recording.

- The duration of the extracellular action potential (AP_ex_) corresponding to the intracellular half-width (see figures 1, 2 and 4) was calculated as the time difference between the minimal and maximal changes in the extracellular waveform.
- The action potential threshold was extracted by numerically calculating the time-dependent second derivative of the membrane potential. The threshold was defined as the point at which the second derivative exceeded 50% of its maximal value.
- The amplitude of the intracellular action potential (AP_amp_) was measured from threshold to the peak of the AP.
- The half-width (AP_hw_) of the intracellular AP was measured as the width at half height of the AP.
- The amplitude of the action potential fast afterhyperpolarization (AHP_f_) was measured from threshold to the peak of the membrane afterhyperpolarization.
- The amplitude of the action potential slow afterhyperpolarization (AHP_s_) was measured from threshold to the second peak of the membrane after-hyperpolarization.
- Input resistance (R_in_) was measured by injecting several hyperpolarizing current steps, recording the response of the membrane potential to these steps, subtracting the resting membrane potential, and calculating R_in_ from linear current-voltage (I–V) curve fitting.
- Time- and voltage-gated anomalous rectification (Sag) reflecting the activation of nonspecific cationic current (I_h_) was measured similarly to input resistance and calculated as the difference between minima of the current-voltage curves and the current-sag curves. Sag ratio was calculated as the ratio between these points.
- Current-frequency (F–I) curves were generated by injecting several depolarizing current steps via the patch pipette and analyzed using exponential curve fitting. The maximal firing rate (F_max_) and the current that induced 63% of F_max_ were extracted from each fit.
- The action potential adaptation ratio was calculated as the ratio between the last and first action potential amplitudes in depolarizing current steps of 50 pA. The existence of rebound firing and the pattern of depolarizing firing in response to injected depolarizing currents were qualitatively evaluated.

Experimental results were consistently obtained from cells from at least 7 rats. All the results for a particular experiment were pooled and displayed as mean ± S.D., unless otherwise stated. A Kruskal-Wallis (KW) one- way analysis of variance by ranks tested equality of the measured cell population based on extracellular and intracellular parameters. A Mann-Whitney-Wilcoxon (MWW) test determined which of the measured extracellular or intracellular parameters divided the GP cell population into three groups.
